# *Edodes Cultured* Extract Regulates Immune Stress During Puberty and Modulates MicroRNAs Involved in Mammary Gland Development and Breast Cancer Suppression

**DOI:** 10.1002/cam4.70277

**Published:** 2024-10-09

**Authors:** Hamed Yasavoli‐Sharahi, Roghayeh Shahbazi, Nawal Alsadi, Samuel Robichaud, Darshan Babu Kambli, Amirhossein Izadpanah, Zhaleh Mohsenifar, Chantal Matar

**Affiliations:** ^1^ Cellular and Molecular Medicine Department, Faculty of Medicine University of Ottawa Ottawa Ontario Canada; ^2^ Department of Pathology University of Montreal Montreal Quebec Canada; ^3^ Department of Stem Cells and Developmental Biology, Cell Science Research Center Royan Institute for Stem Cell Biology and Technology, ACECR Tehran Iran; ^4^ Department of Pathology School of Medicine, Shahid Beheshti University of Medical Sciences Tehran Iran; ^5^ School of Nutrition Sciences, Faculty of Health Sciences University of Ottawa Ottawa Ontario Canada

**Keywords:** breast cancer, LPS, mammary glands development, microbiome and dysbiosis, microRNAs, prebiotics, pro‐inflammatory cytokines, tumor development

## Abstract

**Background:**

Immune stressors, such as lipopolysaccharides (LPS), profoundly affect microbiota balance, leading to gut dysbiosis. This imbalance disrupts the metabolic phenotype and structural integrity of the gut, increasing intestinal permeability. During puberty, a critical surge in estrogen levels is crucial for mammary gland development. However, inflammation originating from the gut in this period may interfere with this development, potentially heightening breast cancer risk later. The long‐term effects of pubertal inflammation on mammary development and breast cancer risk are underexplored. Such episodes can dysregulate cytokine levels and microRNA expression, altering mammary cell gene expression, and predisposing them to tumorigenesis.

**Methods:**

This study hypothesizes that prebiotics, specifically *Lentinula edodes* Cultured Extract (AHCC), can counteract LPS's adverse effects. Using BALB/c mice, an acute LPS dose was administered at puberty, and breast cancer predisposition was assessed at 13 weeks. Cytokine and tumor‐related microRNA levels, tumor development, and cancer stem cells were explored through immunoassays and qRT‐PCR.

**Results:**

Results show that LPS induces lasting effects on cytokine and microRNA expression in mammary glands and tumors. AHCC modulates cytokine expression, including IL‐1β, IL‐17A/F, and IL‐23, and mitigates LPS‐induced IL‐6 in mammary glands. It also regulates microRNA expression linked to tumor progression and suppression, particularly counteracting the upregulation of oncogenic miR‐21, miR‐92, and miR‐155. Although AHCC slightly alters some tumor‐suppressive microRNAs, these changes are modest, highlighting a complex regulatory role that warrants further study.

**Conclusion:**

These findings underscore the potential of dietary interventions like AHCC to mitigate pubertal LPS‐induced inflammation on mammary gland development and tumor formation, suggesting a preventive strategy against breast cancer.

## 1 Introduction

Gut microbiota homeostasis can be significantly affected by dietary choices and inflammatory stressors, such as lipopolysaccharides (LPS) [1]. Furthermore, an imbalanced population of gut microbiota not only triggers changes in gut phenotypes but also contributes to compromised gut barrier integrity, referred to as leaky gut [2]. As a result of this process, the gut's permeability is compromised, allowing bacteria or their byproducts such as LPS to breach the epithelial barrier and initiate a systemic immune response [3, 4]. The adverse effects of dysbiosis can ultimately lead to dysregulation of gene expression in the mammary glands and increase the risk of breast cancer [5].

**FIGURE 1:**
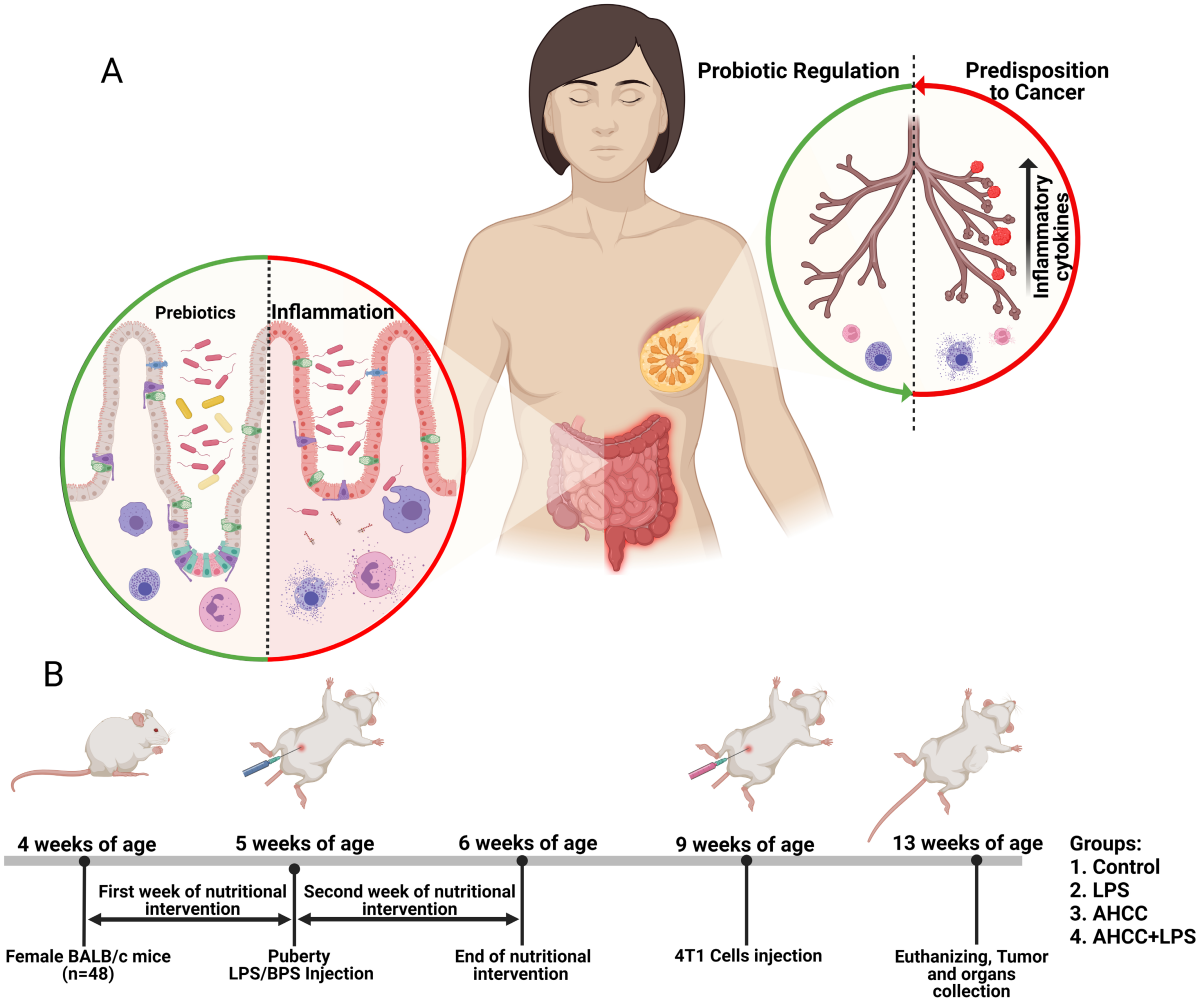
Gut‐immune interactions, mammary gland development, and experimental design. (A) The interplay between gut and microbiota and mammary gland development early in life and potential breast cancer later in life. An activated immune system produces pro‐inflammatory factors, such as TNFα, IL‐1β, and Cox‐2. Given that the gut immune system is a part of mucosal‐associated lymphoid tissue (MALT), exposure to inflammatory agents or immune stressors during critical periods, such as puberty, has the potential to disrupt the normal development of mammary glands and alter their gene expression. This can ultimately contribute to the acquisition of a CSC phenotype and the development of breast cancer. (B) Study design showing the experimental timeline, groups, dietary intervention, LPS injection, and 4T1 cells injection.

LPS plays a critical role as an inflammatory trigger, potentially linking it to cancer susceptibility, particularly in distant organs like the mammary glands [6, 7, 8]. This link is supported by the interconnected mucosal immune system theory, which proposes immunological communication between the gut and other mucosal sites [9]. Obesity‐associated gut microbiota alterations are linked to elevated IL‐6 levels, which activate CD4^+^ T helper cells (Th17) and macrophages, increasing the production of inflammatory mediators, such as TNFα, IL‐1β, and Cox‐2 [10, 11, 12]. Th17 cells, crucial immune modulators, are known to migrate from the gut to other mucosal‐associated lymphoid tissue (MALT) network organs, including the breast, where they can trigger inflammation in response to LPS or pathogens by releasing cytokines, such as IL‐17 and IL‐23 [13, 14, 15, 16]. Additionally, inflammation during key developmental stages, such as puberty, triggers not only Th17 cells but also macrophages to produce excessive inflammatory mediators. These mediators can alter the gene and microRNA expression in mammary stem cells, leading to the development of a cancer stem cell (CSC) phenotype and potentially contributing to breast cancer onset (Figure 1A) [17, 18, 19].

The maturation of breast tissues consists of two phases, including embryonic and pubertal stages [20, 21]. The mammary glands fully mature during puberty, undergoing a significant transformation from terminal end buds into a complex, branched ductal network through branching morphogenesis [22]. Disruptions from inflammatory agents or immune stressors during this phase can compromise cell functions, raising the risk of developmental anomalies and subsequent cancer susceptibility [23, 24, 25]. The research underscores the gut microbiome's vital influence on mammary gland development and its indirect role in cancer risk, with diet during puberty being a significant environmental factor [26, 27, 28]. For instance, a diet rich in n‐3 polyunsaturated fatty acids has been shown to reduce terminal end bud numbers, reflecting healthier mammary gland development [29].

The developmental stage significantly shapes mammary cell fate, with mammary stem cells, particularly during puberty, playing a key role in forming the ductal tree through extensive branching. These highly proliferative cells may acquire early signs of malignancy during this period, with the potential for reactivation under certain conditions, such as inflammation, leading to a cancerous stem cell state [30, 31, 32]. In breast cancer, a small subpopulation of cells termed cancer stem cells (CSCs) possesses a unique ability for self‐renewal and differentiation into diverse cell types. These cells can generate the majority of a tumor's mass, initiate metastasis, and exhibit resistance to chemotherapy, following similar developmental pathways as normal stem cells to proliferate and differentiate [33, 34]. Our ongoing research suggests that inflammation during puberty may increase the likelihood of stem cells implicated in mammary gland formation acquiring a long‐lasting epigenetic signature that could heighten susceptibility to breast cancer later in life (Unpublished Data).

MicroRNAs (miRNAs), short non‐coding RNA sequences about 22 nucleotides long, play a crucial role in gene regulation by binding to the 3′UTR of mRNAs, affecting mRNA expression through processes like cleavage or suppression [35]. Gut microbiota and miRNAs interact in intestinal epithelial cells, leading to the activation of key signaling pathways and modulation of gene expression [36]. For example, miR‐184 notably increases during pubertal mammary development [37]. Disruptions in miRNA balance during key developmental stages could predispose to breast cancer later in life. Moreover, numerous miRNAs, such as miR‐10b, miR‐20, and up to miR‐575, have been identified as influential in the progression of breast and gastric cancers, with specific miRNAs like miR‐145 being downregulated and miR‐155 upregulated in breast cancer [38, 39, 40, 41, 42, 43]. miR‐184 notably curbs metastasis in triple‐negative breast cancer by targeting the Akt/mTORC1 pathway [44].

Prebiotics and probiotics play a crucial role in gut health by maintaining immune balance, influencing metabolism, and controlling pathogens [45, 46]. Their consumption during puberty can counteract LPS‐induced dysbiosis [47]. These compounds not only affect gut microbiota but also modulate miRNAs, altering transcriptome profiles including miR‐192 and miR‐215 expression [48]. They are beneficial in cancer prevention and treatment by impacting miRNA expression, which influences key pathways in metastasis and carcinogenesis [49, 50]. Specifically, *Lentinula edodes* Cultured Extract (AHCC) acts as a TLR4 antagonist, reducing inflammation and the metastatic traits of breast cancer by suppressing ROS production and VE‐cadherin phosphorylation [51].

In this study, mice exposed to the inflammatory agent LPS during puberty were subsequently treated with AHCC, a prebiotic derived from shiitake mushrooms known for its immunomodulatory effects, including inhibition of the TLR4 pathway [52]. This research aimed to explore how AHCC could mitigate the long‐term impacts of LPS exposure on breast cancer development. We examined cytokines, microRNA expression patterns in mammary glands and tumors, tumor progression, and cancer stem cell spheres (CSCS). Our findings suggest that while LPS exposure during puberty may promote breast carcinoma, concurrent AHCC administration could offer protective benefits against breast cancer, highlighting potential preventive strategies for mammary‐related diseases.

## 2 Materials and Method

### 2.1 Cell Line

Murine breast cancer 4T1 cell lines obtained from the American Type Culture Collection (ATCC, Manassas, VA, USA) were cultured in Roswell Park Memorial Institute (RPMI, Gibco, Gaithersburg, MD), supplemented with 10% fetal bovine serum (Gibco, Gaithersburg, MD) and 1% penicillin/streptomycin mixture in a humidified 5% CO_2_ atmosphere at 37°C until reaching 80% confluence. Cells were collected using 0.25% trypsin–EDTA solution.

### 2.2 Animals

Female BALB/c mice, 3 weeks old and weighing 13–17 g, were obtained from Charles River (Montreal, CA). They were maintained in plastic cages, four per cage, under a 12‐h light–dark cycle at 22 ± 2°C and 55 ± 2% humidity. A standard balanced diet was provided. All procedures adhered to the Canadian Council on Animal Care's guidelines and received approval from the University of Ottawa's Animal Care Committee (Protocol HSe‐3418).

### 2.3 Study Design

The study investigated the prolonged effects of pubertal LPS exposure and prebiotic (AHCC) intake on mammary gland development and tumor progression in 48 mice. Puberty onset was marked by the first vaginal opening. Mice were divided into two primary groups: one receiving AHCC‐infused water aimed at a 2 g/kg dosage based on average consumption and another receiving regular water. This dosage was selected as it is equivalent to the recommended human dose provided by the manufacturer and supported by previous studies, ensuring the translational relevance of the findings [53, 54, 55]. Daily water consumption was monitored to estimate and ensure consistent AHCC dosage. At 5 weeks of age, each group was subdivided: one subgroup received an intraperitoneal LPS injection (1.5 mg/kg body weight, *Escherichia coli* O26 LPS dissolved in sterile PBS at 0.2 mg/mL) and the other received a sterile PBS injection, creating four groups: control, LPS, AHCC, and AHCC with LPS (AHCC + LPS). After 1 week of specific dietary intervention postinjection, a standard regimen was resumed. By the ninth week, 4T1 cells suspended in serum‐free RPMI were subcutaneously injected into the abdominal mammary glands (2000 cells in 0.1 mL). Four weeks postinjection, tumors, mammary glands, and blood were collected for analysis posteuthanasia. Tumor size was measured using calipers, with volume calculated by *V* = 0.5 × *d* ^2^ × *D* [56]. AHCC, provided in powder form by Amino Up Co., Ltd., Sapporo, Japan, the original manufacturer and distributor, was shipped specifically for this experiment to ensure consistency and quality (Figure 1B).

### 2.4 Sphere Formation

Tumor samples were surgically removed, sectioned into small pieces, and digested using gentleMACS Dissociator (Miltenyi Biotec., Germany) following the manufacturer's protocol. The digested suspension was filtered through a 70‐μm‐cell strainer and washed with serum‐free RPMI. Dissociated cells were then seeded in ultra‐low attachment 96‐well plates (Millipore Sigma, Oakville, Canada) at a density of 1000 cells per 0.2 mL per well. The growth medium was DMEM‐F12 (Invitrogen, USA) supplemented with 10 ng/mL EGF, 20 ng/mL BFGF, 5 μg/mL insulin, 1 mM sodium pyruvate, 0.5 μg/mL hydrocortisone, and 0.05 mg/mL penicillin/streptomycin. Spheres were counted after 24 h.

### 2.5 Real‐Time Quantitative Reverse Transcription PCR (RT‐qPCR)

A tissue segment from the mammary glands and tumor was preserved in RNAlater stabilization solution (Invitrogen, USA) at 4°C for 1 day, then stored long term at −80°C. Total RNA was isolated from these samples using the miRNeasy mini kit (Qiagen, Toronto, Canada). RNA concentration was measured using NanoDrop 2000 (Thermo Scientific, Waltham, MA, USA). For miRNA level measurement, cDNA was synthesized using the miRCURY LNA RT Kit (Qiagen, Toronto, Canada). The expression levels of microRNAs Let‐7a, Let‐7c, miR‐21, miR‐34a, miR‐92, miR‐125, miR‐140, miR‐145, miR‐155, miR‐181, miR‐184, miR‐188, miR‐200c, miR‐205, miR‐223, and miR‐425 were quantified by RT‐qPCR using the miRCURY LNA miRNA PCR (Qiagen, Toronto, Canada) on a CFX 384 real‐time PCR detection system (Bio‐Rad, Laboratories, Hercules, CA, USA). The expression of miRNAs was normalized to the reference gene SNORD65 (mmu). Relative expression levels of miRNAs were calculated using the ∆∆CT method.

### 2.6 Multiplex Luminex Immunoassay

Levels of IL‐1β, IL‐6, IL‐10, IL‐17A, IL‐17F, IL‐23, TGF‐β1, TGF‐β2, and TGF‐β3 in mammary glands and tumor tissues were analyzed from samples excised using surgical blades (Fisher Scientific, Toronto, Canada) and placed in microtubes containing lysis buffer (20 mM/L Tris–HCl at pH 7.5, 150 mM/L NaCl, 0.05% Tween‐20) and a protease inhibitors cocktail (AEBSF, Hydrochloride, Millipore Sigma, Oakville, Canada). After vortexing and incubating at 4°C for 20 min, samples were homogenized and centrifuged at 14,000 g for 10 min at 4°C to collect the supernatant. Total protein concentrations in the lysates were determined using the BCA method with the Pierce BCA Protein Assay Kit (Thermo Fisher Scientific, Toronto, Canada) and normalized to 2 mg/mL. The multiplex assay was conducted using the mouse Th17 Panel Magnetic, MTH17MAG‐47K, and the TGF‐ΒMAG‐67K MAG bead kit (Millipore Sigma, Burlington, USA), following the manufacturer's instructions. Results were obtained using a MAGPIX System (Millipore Sigma, Burlington, USA).

### 2.7 Statistical Analysis

Statistical analysis was performed using GraphPad Prism software (GraphPad Software Inc., San Diego, CA, USA). Data distribution was evaluated by the Shapiro–Wilk test. Differences in mean values among experimental groups were analyzed using a two‐way analysis of variance (ANOVA), followed by Tukey's post hoc test. Results were presented as mean ± SEM, with a *p* value below 0.05 considered statistically significant. Pathway analysis and miRNA roles were examined using the R programming language with the enrichR package (v 3.0) and miRPathDB (V 2.0).

## 3 Results

### 3.1 Measuring the Modulatory Effects of AHCC and LPS on Tumor Volume, Mass, and Cancer Stem Cell Spheres Abundance

**FIGURE 2:**
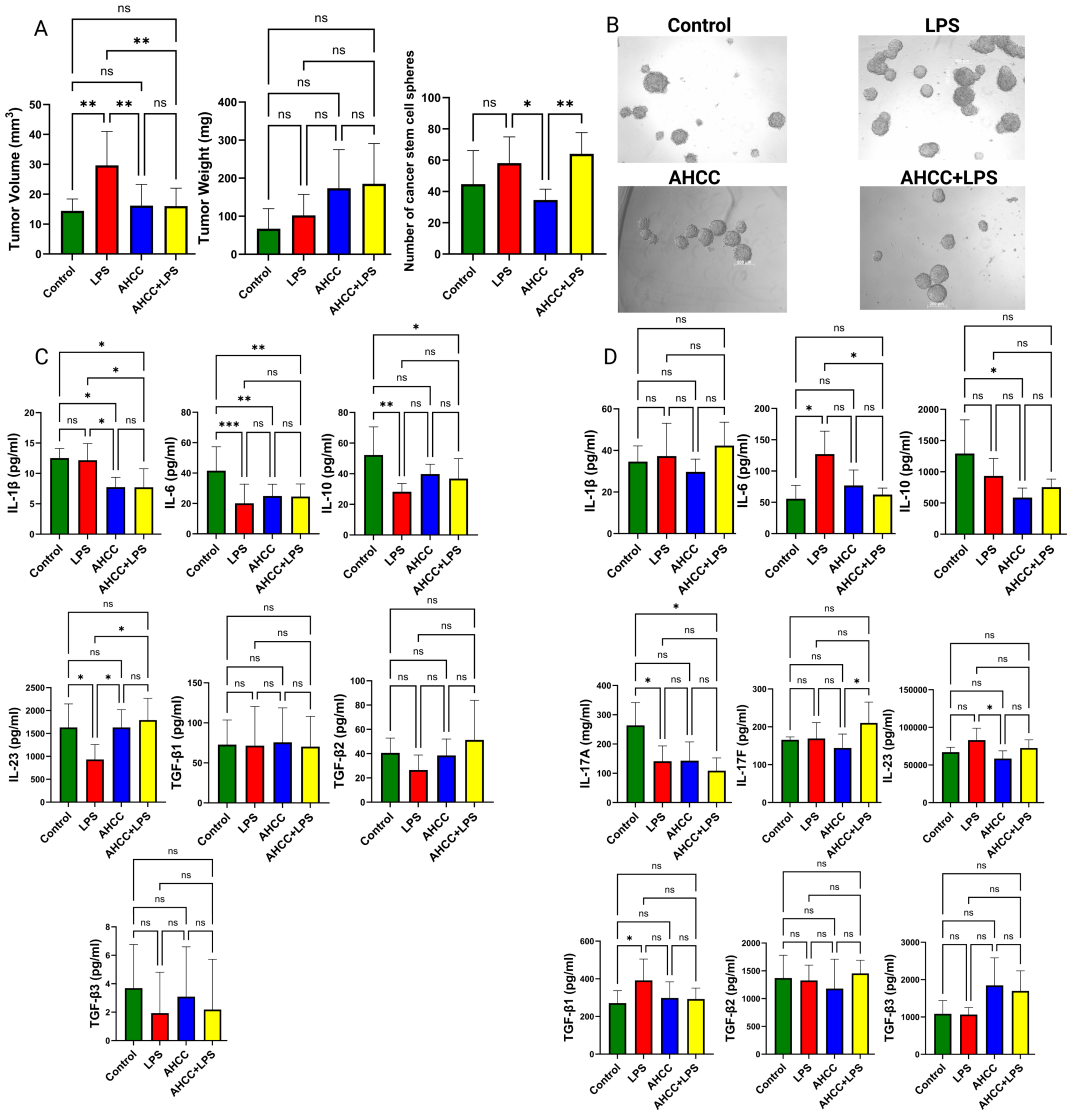
Effects of AHCC and LPS on tumor characteristics. (A) represent the tumor volume, weight, and CSCs. (B) Indicate the number and size of CSCS in the treatment groups. (C) Concentrations of IL‐1β, IL‐6, IL‐10, IL‐23, TGF‐β1, TGF‐β2, and TGF‐β3 in the tumor samples. (D) Concentrations of IL‐1β, IL‐6, IL‐10, IL‐17A, IL‐17F, IL‐23, TGF‐β1, and TGF‐β3 in the mammary gland samples. The mice were randomly assigned to receive either AHCC (2 g/kg BW/d) in their drinking water or plain drinking water for 2 weeks, 1 week before, and 1 week after the pubertal LPS injection. Two‐way ANOVA and Tukey's post hoc tests were used to compare groups. All values are mean ± SEM. *p < 0.05, **p < 0.01, ***p < 0.001, and ****p < 0.0001.

In the initial phase, we examined the treatment's impact on tumor characteristics, focusing on tumor volume, mass, and the abundance of cancer stem cell spheres (CSCS) in each sample. Remarkably, mice treated with LPS exhibited a significant increase in tumor volume compared to both the control and AHCC+LPS groups. In contrast, AHCC treatment did not result in significant differences in tumor volume compared to the control group. Additionally, tumor weight was elevated in all groups relative to the control, although these differences were not statistically significant (Figure 2A).

Analysis of the CSCS count revealed higher numbers in both the LPS and AHCC+LPS groups compared to the control, although these differences were not statistically significant. The AHCC group showed a trend toward lower CSCS abundance compared to the control, LPS, and AHCC+LPS groups, but again, this difference was not statistically significant (Figure 2A). Figure 2B illustrates the CSCS number and size across the experimental groups.

### 3.2 Evaluating AHCC's Potential in Modulating Cytokine Responses to LPS in Tumor Context

Our main goal in this study was to investigate the levels of inflammatory cytokines, including IL‐1β, IL‐6, IL‐10, IL‐23, IL‐17A, and IL‐17F in the tumors (Figure 2C). First, we were unable to detect any presence of IL‐17A and IL‐17F, the main cytokines produced by Th17 [57], in the tumor samples. In terms of IL‐1β expression, there was no significant difference between the LPS group and the control group. However, IL‐1β expression was lower in the AHCC group compared to the control group. Furthermore, the AHCC+LPS group displayed a lower IL‐1β level compared to the LPS groups. Notably, there was no significant difference between the AHCC and AHCC+LPS groups.

Our investigation into IL‐6 revealed reduced levels in all experimental groups compared to the control group. Interestingly, no significant difference was observed between the LPS and AHCC+LPS groups.

Regarding IL‐10, we observed decreased expression levels in all tumor groups compared to the control group. However, the difference was statistically significant only in the LPS group compared to the control group. Moreover, there were no statistically significant differences between the AHCC+LPS and LPS groups.

IL‐23 showed a significant reduction in the LPS group, while its presence remained unchanged in the AHCC group compared to the control group. Furthermore, the expression level of IL‐23 in the LPS group was significantly lower than that in the AHCC+LPS group.

Alongside our examination of inflammatory cytokines, we investigated the expression patterns of TGF‐β1, TGF‐β2, and TGF‐β3. For TGF‐β1, consistent expression was observed across all study groups, with no significant differences identified. As for TGF‐β2, a decrease was noted in its level in the LPS group. Meanwhile, although TGF‐β3 levels declined in both the LPS and AHCC+LPS groups, the differences were not statistically significant (Figure 2C).

### 3.3 Evaluating AHCC's Potential in Modulating Cytokine Responses to LPS in Mammary Gland Context

We assessed the expression levels of inflammatory cytokines in normal mammary glands adjacent to the tumors, focusing on IL‐1β, IL‐10, IL‐23, IL‐17A, and IL‐17F (Figure 2D). Interestingly, we observed a reduction trend in IL‐1β expression in the AHCC group compared to the AHCC+LPS group; however, no statistically significant differences were found between the groups.

Regarding IL‐6, we noted higher expression levels in the LPS group compared to the control group. Interestingly, AHCC attenuated the effect of LPS, as evidenced by lower IL‐6 expression in the AHCC+LPS group compared to the LPS group.

IL‐10 demonstrated reduced expression levels in all groups, with statistically significant differences observed only in the AHCC group compared to the control group.

ILcd among the other groups (Figure 2D). As for IL‐17F, we found increased expression in the AHCC+LPS group compared to AHCC, but no other comparisons yielded statistically significant differences.

Turning to IL‐23, an increase was observed in the LPS group compared to both the control and AHCC groups; however, the only statistically meaningful comparison was between AHCC and LPS. Moreover, IL‐23 expression in the AHCC+LPS group did not significantly differ from that in the LPS group.

Regarding TGF‐β1, higher expression levels were observed in the LPS group compared to the control group. No significant differences were found between the AHCC and AHCC+LPS groups compared to the control group. Furthermore, TGF‐β1 expression in the LPS group was higher than that in the AHCC+LPS group; however, the differences were not statistically significant.

For TGF‐β2, we found that its expression level was decreased in the AHCC group, but none of the comparisons reached statistical significance.

TGF‐β3 exhibited no difference in the LPS group compared to the control group. In contrast, TGF‐β3 expression demonstrated an increase in both the AHCC and AHCC+LPS groups compared to the control and LPS groups; however, none of the comparisons were statistically significant (Figure 2D).

### 3.4 Investigating the miRNA Regulatory Effects of AHCC on LPS‐Induced MicroRNA Patterns in Tumors

**FIGURE 3:**
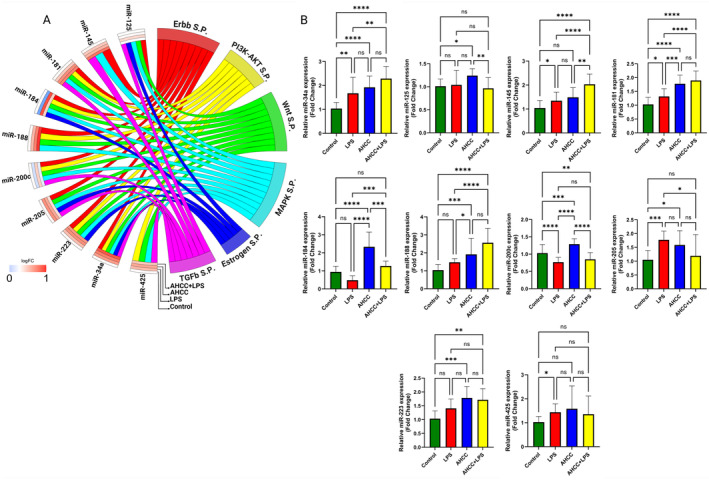
Effects of AHCC and LPS on microRNA Expression in the Tumors. (A) The enrichment gene ontology plot indicates the role of microRNAs in the important pathways affecting tumor development. The left side of the plot is the heatmap, showing the log2 expression of the selected microRNAs. (B) The relative expression of candidate microRNAs, including let‐7a, let‐7c, miR‐21, miR‐34a, miR‐92, miR‐125, miR‐140, miR‐155, miR‐181c, miR‐200c, and miR‐425 in the tumor of adult mice 4 weeks after 4T1 cells injection. The mice were randomly assigned to receive either AHCC (2 g/kg BW/d) in their drinking water or plain drinking water for 2 weeks, 1 week before, and 1 week after the pubertal LPS injection. Two‐way ANOVA and Tukey's post hoc tests were used to compare groups. All values are mean ± SEM. *p < 0.05, **p < 0.01, ***p < 0.001, and ****p < 0.000.

In the next step, we investigated the expression levels of microRNAs implicated in tumor progression (Figure 3A). The panel of microRNAs chosen for this study was based on their well‐documented roles in cancer development, progression, and their involvement in maintaining homeostasis and mammary gland development. The specific microRNAs analyzed included Let‐7a, Let‐7c, miR‐21, miR‐34a, miR‐92, miR‐125, miR‐140, miR‐155, miR‐181c, miR‐200c, and miR‐425 (Figure 3B).

Let‐7a exhibited no significant changes in the LPS group compared to the control group. In the AHCC and AHCC+LPS groups, an increase in Let‐7a was observed compared to the control group; however, these comparisons were not statistically significant. Moreover, a significant upregulation was observed in the AHCC+LPS group compared to the LPS group.

Let‐7c demonstrated a decrease in expression in the LPS group relative to the control group. In the AHCC group, the expression level slightly decreased, but the difference was not statistically significant. Moreover, Let‐7c showed a significant increase in the AHCC+LPS group compared to both the control and LPS groups.

Notably, miR‐21, miR‐92, and miR‐155 exhibited similar trends, with significant upregulation in the LPS group compared to the control, AHCC, and AHCC+LPS groups, indicating that AHCC may mitigate the effect of LPS. Although the expression levels of these three microRNAs were slightly higher in the AHCC and AHCC+LPS groups compared to the control, these differences were not statistically significant.

Regarding miR‐34a and miR‐181c, a reduction in expression was observed in the LPS group compared to the control group. Specifically, for miR‐181c, when compared between the LPS and AHCC+LPS groups, a higher expression level was found in the AHCC+LPS group, suggesting that AHCC may mitigate the effects of LPS.

For miR‐125 and miR‐140, no significant changes were observed in the LPS group compared to the control group. However, their expression levels were increased in the AHCC group compared to the control group. miR‐125 showed the highest expression level in the AHCC+LPS group compared to all other groups, while miR‐140 tended to be lower in the AHCC+LPS group compared to all other groups, with a significant difference observed only in the comparison between AHCC and AHCC+LPS groups.

Surprisingly, miR‐200c showed significantly increased expression in all groups compared to the control group. There were no significant differences between the LPS and AHCC+LPS groups, but the AHCC group displayed the highest expression level for miR‐200c among all groups.

Concerning miR‐425, it exhibited increased expression only in the AHCC+LPS group compared to all other groups. No differences were observed between the LPS and AHCC groups compared to the control group (Figure 3B).

### 3.5 Investigating the Regulatory Effects of AHCC on LPS‐Induced MicroRNA Patterns in Mammary Glands

**FIGURE 4:**
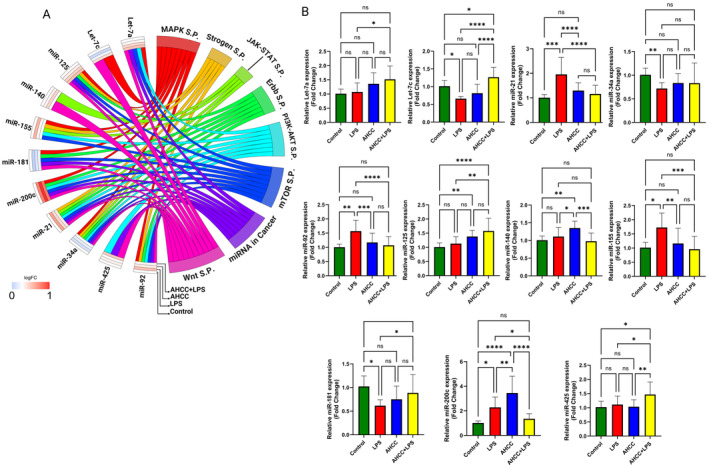
Effects of AHCC and LPS on microRNA expression in the adjacent mammary glands. (A) The enrichment ontology plot indicating the role of microRNAs in the important pathways affecting tumor development. The left side of the plot is the heatmap, showing the log2 expression of the selected microRNAs. (B) The relative expression of candidate microRNAs, including miR‐34a, miR‐125, miR‐145, miR‐181, miR‐184, miR‐188, miR‐200c, miR‐205, miR‐223, and miR‐425, in the mammary glands of adult mice 4 weeks after 4T1 cells injection. The mice were randomly assigned to receive either AHCC (2 g/kg BW/d) in their drinking water or plain drinking water for 2 weeks, 1 week before, and 1 week after the pubertal LPS injection. Two‐way ANOVA and Tukey's post hoc tests were used to compare groups. All values are mean ± SEM. *p < 0.05, **p < 0.01, ***p < 0.001, and ***p < 0.0001.

Similarly, we explored the expression levels of several microRNAs that play crucial roles in mammary gland development and are known to affect tumor growth. The candidate microRNAs included miR‐34a, miR‐125, miR‐145, miR‐181c, miR‐184, miR‐188, miR‐200c, miR‐205, miR‐223, and miR‐425 (Figure 4A). This selection was made to capture the microRNAs most likely to reveal insights into the effects of AHCC on mammary gland development and tumor progression. Remarkably, we observed a consistent pattern of increased expression for microRNAs miR‐34a, miR‐145, miR‐181c, miR‐188, miR‐223, and miR‐425 in all treatment groups (LPS, AHCC, and AHCC+LPS) compared to the control group (Figure 4B).

Specifically, miR‐34a and miR‐181c showed significant upregulation in all treatment groups relative to the control group, with the AHCC+LPS group exhibiting the highest expression levels among all treatment groups. For miR‐188 and miR‐223, although there were increases in expression in the LPS group compared to the control, these differences did not reach statistical significance. On the other hand, miR‐145 and miR‐425 showed statistically significant increases in expression in the LPS group compared to the control group.

Regarding miR‐125, we observed increased expression in the AHCC group compared to the control group, while its expression remained unchanged in the LPS group compared to the control and AHCC+LPS groups.

Furthermore, miR‐184 and miR‐200c exhibited decreased expression in the LPS group compared to the control group. In contrast, their expression levels were higher in the AHCC group compared to the control group. Notably, miR‐184 showed higher expression in the AHCC+LPS group compared to the LPS group, indicating that AHCC not only mitigated the effect of LPS but also promoted the expression of miR‐184. Additionally, miR‐200c exhibited slightly lower expression in the AHCC+LPS group compared to the control group, and higher expression compared to the LPS group, although these differences were not statistically significant.

Finally, miR‐205 displayed higher expression in the LPS group compared to both the control and AHCC+LPS groups. Although miR‐205 also showed higher expression in the AHCC group compared to the control and AHCC+LPS groups, these differences were not statistically significant (Figure 4B).

## 4 Discussion

Probiotics and prebiotics, part of the “Biotics” family, play a critical role in modulating gut microbial ecosystems and reducing systemic inflammation, with known chemopreventive effects against various cancers [52, 58, 59, 60]. Previous studies have demonstrated the influence of probiotics on mammary carcinoma, emphasizing the gut microbiome's role at both local and distant sites [59, 61, 62]. The gut microbiome's complex interaction with breast cancer involves immunological cross‐talk between the gut and extra‐intestinal sites like the mammary glands, facilitated by the mucosal immune system and the MALT system, which transports microorganisms via immune cells [9, 63]. Disruptions in Th17 cell regulation can impact lymphocyte activities and deregulate TGF‐B signaling, highlighting the significant role of prebiotics in influencing cytokine secretion and gene expression, potentially affecting tumor development [9, 64, 65].

In this study, the effects of LPS and AHCC treatment on mammary glands, tumor characteristics, inflammatory cytokines, and microRNA expression patterns in mice were systematically examined. A significant increase in tumor volume was observed following LPS treatment, consistent with findings that bacterial LPS can activate inflammatory pathways contributing to tumor development [66]. This phenomenon aligns with the well‐established role of chronic inflammation, driven by elevated LPS levels, in facilitating tumor progression [67]. Interestingly, the combination of AHCC and LPS mitigated the effects of LPS, implying that AHCC possesses anti‐inflammatory properties [68]. The stable tumor volume in AHCC‐treated mice suggests its immunomodulatory rather than tumor‐suppressive action. Increases in tumor weight across all groups suggest that complex factors beyond LPS or AHCC treatment influence tumor weight. These include tumor density, necrosis, stromal content, and immune cell infiltration, necessitating further investigation to precisely identify these factors.

Cancer stem cell (CSC) spheres have become a focus due to their role in therapy resistance, metastasis, and tumor recurrence [69]. An increase in CSCs in the LPS and AHCC+LPS groups suggests that LPS may interact with pathways influencing stemness during critical activity in the pubescent mammary gland, as supported by studies showing LPS‐induced stemness in cancer cells [70]. Although the reduction in CSCs in the AHCC group was not statistically significant, further research is needed to assess whether AHCC can affect CSC dynamics in the presence of LPS‐induced stemness, as depicted in Figure 3D.

The role of cytokines in tumorigenesis, particularly within an inflammatory microenvironment, is critical for tumor development and progression [71]. This study evaluated the response of specific cytokines in tumor samples and mammary glands to LPS and AHCC treatments. AHCC's modulation of cytokine levels, notably the reduced expression of IL‐1β in tumors and mammary glands treated with AHCC, aligns with its known immunomodulatory properties [72, 73]. Additionally, AHCC's co‐administration with LPS seemed to mitigate LPS‐induced inflammation in tumors, suggesting its potential as an anti‐inflammatory agent in cancer contexts. The multifaceted role of IL‐6 in cancer pathogenesis is underscored by its association with poor survival in breast cancer patients [74]. In this study, although no significant differences were noted between the control and LPS‐treated tumors in IL‐6 levels, a reduction in IL‐6 with AHCC treatment suggests a potential therapeutic benefit. IL‐10, known for its dual tumor‐promoting and suppressive effects, showed reduced levels across all groups, particularly with LPS treatment, indicating an inflammatory response [75]. Notably, both AHCC‐treated groups displayed lower IL‐10 levels, suggesting that AHCC may influence cytokine‐mediated immune responses in mammary glands [76]. Additionally, IL‐23, a pro‐inflammatory cytokine critical for Th17 cell differentiation, was increased with LPS administration, with only a slight modulation by AHCC [77, 78, 79]. This observation raises questions about AHCC's effectiveness in altering IL‐23 levels within the mammary gland environment.

In the tumor setting, IL‐23 expression decreased following LPS administration, which contrasts with the expected inflammatory response, suggesting a unique modulation of the IL‐23 pathway in breast tumor cells exposed to LPS. Notably, IL‐17A and IL‐17F were absent in tumor samples, despite their roles in promoting inflammation‐driven tumorigenesis [80]. This decrease in IL‐23 may explain the lack of IL‐17 cytokines, though IL‐23's tumor‐promoting effects may still operate independently of IL‐17 [81]. Tumor‐associated macrophages, the primary source of IL‐23 in the tumor microenvironment, have been linked to the immunosuppressive properties of IL‐23. A recent study revealed that a subset of tumor‐infiltrating regulatory T (Treg) cells exhibits a suppressive phenotype mediated through Il23r, suggesting that reducing IL‐23 could diminish its tumor‐promoting effects by stabilizing an effector cell program [82].

The TGF‐β family, crucial for cell growth, immune responses, and tissue repair, showed stable TGF‐β1 expression levels across all groups in our breast cancer study, aligning with its known dual role in tumor dynamics [83]. Conversely, TGF‐β2 levels declined in all groups except in the AHCC+LPS group, where it remained stable, suggesting that AHCC may counteract LPS effects and offer therapeutic benefits by modulating immune recognition and response to tumors [84].

Despite the decrease in TGF‐β3 expression in the LPS and AHCC+LPS groups, the changes were not statistically significant. TGF‐β3 is involved in EMT and metastasis, suggesting AHCC's potential to modulate cytokine activity and influence inflammation and tumor progression [85]. In mammary glands, TGF‐β1 levels increased with LPS but normalized with AHCC co‐administration, suggesting AHCC's protective effects against inflammatory damage [86, 87]. The stable TGF‐β2 levels in the AHCC group and the increase in TGF‐β3 in AHCC‐treated groups underscore AHCC's potential impact on these cytokines, crucial for tissue repair and mammary gland development [88].

MicroRNAs (miRNAs) are posttranscriptional regulators crucial in cellular processes, such as differentiation, proliferation, and apoptosis, and their dysregulation can significantly affect tumor progression and metastasis [89, 90]. In this study, we investigated key miRNAs linked to tumor progression, including let‐7a/c, miR‐21, miR‐92, miR‐140, and miR‐155, as well as miRNAs associated with mammary gland development, such as miR‐145, miR‐184, miR‐188, miR‐205, and miR‐223, under the influence of LPS and AHCC. We also analyzed the expression patterns of miR‐34a, miR‐125, miR‐181c, miR‐200c, and miR‐425, which play roles in both mammary gland development and tumor progression.

Notably, the upregulation of Let‐7a in the AHCC and AHCC+LPS groups, known to suppress tumor growth and metastasis [91], suggests AHCC's potential protective effect. Similarly, Let‐7c, associated with tumor‐suppressive effects in breast cancer, showed a significant increase in the AHCC+LPS group, highlighting AHCC's role in modulating miRNA expression [92].

Upregulation of miR‐21, miR‐92, and miR‐155 was observed in the LPS group, and miRNAs are known to promote tumor growth and inhibit apoptosis across various cancers [93, 94, 95]. Notably, their expression was mitigated by AHCC, suggesting its potential role in reducing tumor‐promoting effects induced by LPS. Conversely, miR‐140, which inhibits cancer stem cell proliferation and migration, showed increased expression uniquely in the AHCC group. However, its levels decreased in the AHCC+LPS group, underlining the complexity of miRNA regulation. MiR‐140 also plays a role in suppressing the Wnt signaling pathway and enhancing the efficacy of doxorubicin treatment [96, 97].

In mammary glands, miR‐145, miR‐188, and miR‐223 were significantly upregulated across all groups, with the highest levels observed in the AHCC and AHCC+LPS groups. MiR‐145 and miR‐188 are key regulators of lipogenesis and fatty acid metabolism, targeting proteins like FOXO1 and PTEN [98, 99, 100]. MiR‐223, known for its role in the EGFR signaling pathway, affects the growth and organization of mammary epithelial cells and ducts, crucial for lactation [101].

MiR‐184, a tumor suppressor, also plays a critical role in mammary gland development by influencing the PI3K/AKT/mTOR pathway and regulating the differentiation of terminal end bud cells into ductal epithelial cells [102]. Its expression was reduced by LPS but increased in the AHCC and AHCC+LPS groups.

Furthermore, miR‐205, integral to the YAP and Wnt signaling pathways, is primarily expressed in the basal cells of mature mammary glands. Disruptions in miR‐205 expression can impact stem cell renewal and the integrity of mammary epithelial cells [103].

We investigated miRNAs affecting both mammary gland development and tumor progression, including miR‐34a, miR‐125, miR‐181c, miR‐200c, and miR‐425. MiR‐34a and miR‐181c, known for their tumor‐suppressive roles by downregulating Bcl‐2 and MAP4K4, were significantly downregulated in the LPS group but showed higher levels with AHCC treatment, suggesting AHCC's role in mitigating LPS‐induced suppression [104, 105, 106]. In mammary glands, both miRNAs were upregulated across all treatment groups, particularly in the AHCC+LPS group, highlighting the nuanced effects of these treatments on miRNA regulation. MiR‐34a, which maintains mammary epithelial cell homeostasis by inhibiting the Wnt pathway through β‐catenin suppression, and miR‐181c, involved in regulating the PI3K‐Akt pathway crucial for mammary cell proliferation and survival, show complex regulatory dynamics [12, 107, 108].

MiR‐125, known for its crucial roles in cell differentiation and the suppression of tumor progression and metastasis through targeting ENPEP, CK2‐α, CCNJ, and MEGF9, showed elevated levels in the AHCC group, suggesting the regulatory influence of AHCC on miR‐125 [109].

MiR‐200c expression increased in all treatment groups, underscoring its role in suppressing tumor proliferation and epithelial‐mesenchymal transition (EMT) by targeting ZEB1 and ZEB2 [110]. Notably, even under immune stress typically induced by LPS, miR‐200c levels rose, indicating a complex interaction within the tumor microenvironment [111]. In mammary glands, reduced miR‐200c expression correlates with duct elongation by inhibiting EMT.

MiR‐425, known to function as an oncogene and tumor suppressor across different cancers, did not show significant changes with either LPS or AHCC treatment [112, 113]. However, its expression notably increased when both treatments were combined, linking miR‐425 to immune response modulation [114].

MicroRNAs critically regulate signaling pathways in mammary gland development and cancer progression, as well as inflammatory responses. For example, the Let‐7 family suppresses IL‐6, inhibiting NF‐κB and STAT3 activation [115]. MiR‐21 and miR‐155 modulate IL‐10 expression, while miR‐92 is crucial for its production [116, 117, 118]. Our findings include a decrease in IL‐10 in the LPS group, highlighting miRNA's role in inflammation regulation.

MiR‐140 represses IL‐6 and IL‐8 via TLR4 regulation [119], and miR‐145 inhibits mTOR and AKT3, affecting IL‐1β and IL‐6 [120]. miR‐188 targets TLR4, reducing inflammatory cytokines through NF‐κB [121]. Conversely, miR‐223, generally anti‐inflammatory by targeting TLR4 and NLRP3, showed a pro‐inflammatory role by influencing IL‐23 in our study [122, 123, 124]. We observed an increase in miR‐223 across all groups, a decrease in IL‐23 in the LPS group, and an increase with AHCC and AHCC+LPS. miR‐205 mitigates LPS effects by reducing inflammatory cytokines like IL‐1β through COMMD1 targeting, while miR‐34a and miR‐181c decrease IL‐6 and IL‐1β by modulating the NF‐κB pathway [125, 126, 127]. MiR‐125 inhibits IL‐6, JAK1, and STAT3, regulating inflammation independently of LPS [128]. Lastly, miR‐425 regulates IL‐1β and TNFα, mitigating inflammation [114]. These findings underscore AHCC's potential therapeutic effects in counteracting LPS‐induced changes in specific miRNAs. Pathological examination of tumors revealed no significant differences in necrosis, vessel formation, or neutrophil infiltration (data not shown). This study demonstrates the intricate role of prebiotics in modulating inflammation, cytokine levels, and miRNA expression, affecting mammary gland development and breast cancer progression. LPS‐induced inflammation during puberty was found to increase tumor size and elevate specific inflammatory cytokines and oncogenic miRNAs, suggesting activation of inflammatory pathways. Notably, AHCC treatment upregulated tumor‐suppressive microRNAs such as Let‐7a/c and reduced oncogenic miRNAs like miR‐21, miR‐92, and miR‐155. AHCC also boosted miRNAs essential for mammary gland development (miR‐145, miR‐188, and miR‐223) and influenced other miRNAs (miR‐34a, miR‐125, miR‐181c, miR‐200c, and miR‐425) linked to both gland function and tumor growth, highlighting its potential therapeutic benefits. These findings support the role of dietary interventions during puberty in shaping mammary gland development and reducing future breast cancer risk. While this study establishes a link between pubertal inflammation and subsequent effects on mammary glands and tumor development, it underscores that such inflammation is not necessarily causative of breast cancer, reflecting the disease's multifactorial nature.

## Author Contributions

**Hamed Yasavoli‐Sharahi:** conceptualization (lead), data curation (lead), formal analysis (lead), methodology (equal), visualization (lead), writing – original draft (equal), writing – review and editing (equal). **Roghayeh Shahbazi:** conceptualization (equal), data curation (equal), writing – original draft (equal), writing – review and editing (equal). **Nawal Alsadi:** methodology (equal), writing – review and editing (supporting). **Samuel Robichaud:** formal analysis (equal), methodology (equal), writing – review and editing (equal). **Darshan Babu Kambli:** writing – review and editing (equal). **Amirhossein Izadpanah:** formal analysis (equal), writing – review and editing (equal). **Zhaleh Mohsenifar:** formal analysis (equal). **Chantal Matar:** funding acquisition (equal), investigation (equal), methodology (equal), resources (equal), writing – review and editing (equal).

## Ethics Statement

The experimental protocol for all animal experiments conducted in this study was approved by the Animal Care Committee of the University of Ottawa on 13 July 2022, under the approval code HSe‐3191. The care, treatment, and maintenance of mice throughout the course of this research adhered strictly to the guidelines set forth by the Canadian Council on Animal Care, ensuring the highest standards of animal welfare. This study did not involve any human subjects.

## Conflicts of Interest

The authors declare no conflicts of interest.

## Data Availability

The data sets used and/or analyzed during the current study are available from the corresponding author upon reasonable request.
